# Investigating sex, race, and geographic disparities in bronchus and lung cancer mortality in the United States: a comprehensive longitudinal study (1999–2020) utilizing CDC WONDER data

**DOI:** 10.1097/MS9.0000000000002387

**Published:** 2024-08-07

**Authors:** Hafsah Alim Ur Rahman, Muhammad Ahmed Ali Fahim, Afia Salman, Sateesh Kumar, Adarsh Raja, Sandesh Raja, Damni Advani, Raja Devendar, Anuva Khanal

**Affiliations:** a Dow Medical College, Dow University of Health Sciences; b Shaheed Mohtarma Benazir Bhutto Medical College Lyari, Karachi, Pakistan; c Dhulikhel Hospital, Dhulikhel, Nepal

**Keywords:** age-adjusted mortality rates, demographics, lung and bronchus cancer

## Abstract

**Background:**

Lung and bronchus cancer has become the leading cause of cancer-related mortality in the United States. Understanding the patterns of mortality is an absolute requirement.

**Methods:**

This study analyzed Lung and Bronchus cancer-associated mortality rates from 1999 to 2020 using death certificate data from the Centers for Disease Control and Prevention Wide-Ranging OnLine Data for Epidemiologic Research (CDC WONDER). Age-adjusted mortality rates (AAMRs), per 100 000 people, and annual percentage change (APCs) were also calculated.

**Results:**

3 599 577 lung and bronchus cancer-related deaths occurred in patients aged younger than 1–85+ years between 1999 and 2020. Overall AAMRs declined from 59.1 in 1999 to 58.9 in 2001 (APC: −0.1364) then to 55.9 in 2005 (APC: −1.4388*) 50.5 by 2010 (APC: −2.0574*) 44.7 by 2014 (APC: −2.9497*) and 35.1 by 2020 (APC: −4.1040*). Men had higher AAMRs than women (overall AAMR men: 61.7 vs. women: 38.3). AAMRs were highest among non-Hispanic (NH) Black or African American (52.7) patients followed by NH White (51.8), NH American Indian or Alaska Native (38.6), NH Asian or Pacific Islander (24.7) and Hispanic or Latino race (20.2). AAMRs varied in region (overall AAMR; South: 52.4; Midwest: 52.3; Northeast: 46.3; West: 39.1). Non-metropolitan areas had a higher AAMR (55.9) as compared to metropolitan areas (46.7). The top 90^th^ percentile states of Lung and Bronchus cancer AAMR were Arkansas, Kentucky, Mississippi, Tennessee, and West Virginia.

**Conclusion:**

An overall decreasing trend in AAMRs for lung and bronchus cancer was seen. Public health measures to regulate risk factors and precipitating events are needed.

## Introduction

Highlights

- Trends in bronchus and lung cancer mortality: The study reveals a significant and consistent decrease in bronchus and lung cancer mortality rates over the course of two decades, from 1999 to 2020.
- Sex disparities: Men consistently experienced higher age-adjusted mortality rates (AAMRs) compared to women, highlighting sex disparities in bronchus and lung cancer mortality.
- Racial and ethnic variations: NH Black or African American individuals had the highest AAMRs among different racial and ethnic groups, followed by NH White, NH American Indian or Alaska Natives, NH Asian or Pacific Islanders and Hispanics populations.
- Geographic disparities: The study found significant variations in AAMRs across different regions of the United States, with the South having the highest AAMRs, followed by the Midwest, Northeast, and Western regions.
- Urban vs. non-metropolitan discrepancies: Non-metropolitan regions were shown to have higher AAMR values than metropolitan areas.

Cancer ranks as the second leading cause of mortality in the United States and is the primary cause of death among individuals under 85 years of age. The COVID-19 pandemic in 2019 led to setbacks in cancer detection and treatment due to closures in healthcare facilities, disruptions in employment and health insurance, and concerns about exposure to COVID-19. The long-term impact of these delays on cancer diagnosis at advanced stages and subsequent mortality rates will be understood gradually over several years. Projections for 2024 anticipate 2 001 140 new cancer cases and 611 720 cancer-related deaths in the United States. Lung cancer stands out as the primary cause of cancer-related mortality in the United States, representing approximately one-fifth of all cancer deaths. Annually, the number of lung cancer deaths surpasses the combined total of deaths from colon, breast, and prostate cancers. About 340 individuals succumb to lung cancer daily, a figure nearly 2.5 times higher than the number of deaths from colorectal cancer, which ranks second in cancer-related mortalities. It is estimated that around 81% of the 125 070 lung cancer deaths projected for 2024 will directly result from cigarette smoking, with an additional 3500 deaths attributed to exposure to second-hand smoke. If classified separately, the ~20 300 lung cancer deaths not linked to smoking would rank as the eighth leading cause of cancer death across sexes^1–3^. The occurrence of lung cancer has shown a consistent decrease since 2006, with a yearly decline of 2.5% in men and 1% in women. This decline commenced later in women and has been less rapid compared to men, likely due to a delayed and slower decline in smoking habits among women, including increases in smoking prevalence among certain birth cohorts^1,4,5^. Understanding the demographic and regional patterns of mortality related to lung and bronchus malignant neoplasms is crucial for identifying high-risk populations and delivering targeted interventions promptly. Thus, we aimed to assess the demographic and regional variances in lung and bronchus malignant neoplasm mortality among U.S. adults from 1999 to 2020.

## Methods

### Study setting and population

To ascertain the mortality rates attributed to Lung and Bronchus cancer the following study made use of death certificate information from The Centers for Disease Control and Prevention Wide-Ranging OnLine Data for Epidemiologic Research (CDC WONDER) database examining cases between 1999 and 2020. The International Classification of Diseases and Related Health Problems, 10th edition (ICD-10), diagnostic codes C34.0, C34.1, C34.2, C34.3, C34.8, and C34.9 were utilized in the aforementioned database to narrow down Lung and Bronchus cancers. Numerous previous studies have examined patterns of mortality related to cancer using this method with data from the District of Columbia, all 50 states, and other states being included. To identify Lung and Bronchus Cancer cases, death records from the Multiple Causes of Death Public Use registry were surveyed with Lung and Bronchus Cancer as either a primary or secondary cause of death. Patients examined in our study were defined as less than one year to more than 85 years at the time of death. Previous studies have also made use of such an age range in patient categorization^6–8^. In addition, a sensitivity analysis considering neoplasms (C00-D48) as the underlying cause of death in the background of Lung and Bronchus cancer-related mortality was also carried out. This study complied with STROBE guidelines and since it utilized de-identified, government-released public use data, no regional institutional review board approval was required.

### Data abstraction

Population size, year, location of death, demographics, geographic division, state state-specific data in addition to urban and rural classification included in the data abstracted. Location of death included homes, hospices, nursing homes, long-term care facilities, and hospitals with demographics referring to age, sex, and race or ethnicity. Furthermore, race was stratified into two types non-Hispanics (NH), defined as individuals who do not have ancestral ties to Spanish-speaking countries, particularly those in Latin America; and Latino or Hispanic, which is defined as a person of Cuban, Mexican, or Puerto Rican, South or Central American, or other Spanish culture or origin, regardless of race. Additionally, the NH race was further sub-grouped into NH White people, NH Black or African American people, NH American Indians or Alaska Native people, and NH Asian or Pacific Island people. The data utilized in the analysis was death certificate reported information and has also been a source for earlier research using the aforementioned database^9^.

The National Center for Health Statistics Urban-Rural Classification Scheme was used to divide the population into categories of urban and rural regions according to the 2013 U.S. Census^10^. Urban areas were subdivided into medium/small metropolitan areas, which had a population between 50 000 and 999 999, and large metropolitan areas, which had a population of one million or more. Rural regions were defined as having less than 50 000 people. Categorization of geographical areas into the Northeast, Midwest, South, and West regions was done via the United States Census Bureau’s criteria^11^.

### Statistical analysis

Trends in Lung and Bronchus Cancer-related mortality were analyzed by calculating mortality rates per 100 000 of the population for both age-adjusted and crude data, along with their respective 95% CIs, during the 1999–2020 period, categorizing them according to year, sex, race/ethnicity, state, urban/rural status.

In the calculation of crude mortality rates, the total number of deaths corresponding to Lung and Bronchus Cancer in a year was divided by the population for that year. For age-adjusted mortality rates (AAMR) calculations, Lung and Bronchus cancer-related deaths in the United States population in 2000 were standardized^12^. The Joinpoint Regression Programme (Version 5.0.2, National Cancer Institute) was utilized to determine annual variations in Lung and Bronchus Cancer-related mortality by calculating annual percent changes (APC), along with their respective 95% CI, in age-adjusted AAMR^13^. This method makes use of log-linear regression models to recognize significant changes in AAMR over time. APCs were considered as increasing or decreasing when the two-tailed t-test indicated that the slope representing changes in mortality deviated significantly from zero. Lastly, a *P* value of *P* less than 0.05 was considered statistically significant.

## Results

Lung and bronchus cancer between the years 1999 and 2020 caused a total of 3 599 577 deaths in patients aged younger than 1–85+ years (Supplementary Table 1, Supplemental Digital Content 1, http://links.lww.com/MS9/A569). AAMRs for lung and bronchus Cancer-related mortalities in the United States stratified according to age groups are given in Table 1 with the 75–84 years age group having the highest AAMR at 361 (29.90% of deaths). Additionally, the age groups of younger than 1 year, 1–4 years, and 5–14 years presented with the lowest AAMR at 0 (0% of deaths) each. Other notable AAMRs include those for the 85+ year and 65–74 year age groups at 338 and 223.6, respectively. The overall AAMR for all age groups emphasizing the age-specific impact of Lung and Bronchus Cancer was revealed to be 53.4. The location of death was recorded for 3 588 558 deaths of which, 33.37% occurred at medical facilities, 13.64% at nursing homes, 7.00% at hospices, 40.83% at home, and 5.15% at other places that could not be included in the aforementioned categories, with the place of death of the remaining 11019 patients being unknown. (Supplementary Table 2, Supplemental Digital Content 1, http://links.lww.com/MS9/A569).

**Table 1:** Demographic characteristics of deaths due to lung cancer among all ages in the USA from 1999 to 2020.

Variable	Lung cancer deaths, *n* (%)	AAMRs (95% CI) per 100 000
Overall population	3 599 577 (100)	48.3 (48.3–48.4)
Sex		
Male	2 009 294 (55.8)	61.7 (61.6–61.8)
Female	1 590 283 (44.2)	38.3 (38.2–38.3)
Census region		
Northeast	666 618 (18.5)	46.3 (46.2–46.5)
Midwest	869 878 (24.2)	52.3 (52.2–52.4)
South	1 440 608 (40.0)	52.4 (52.3–52.5)
West	622 473 (17.3)	39.1 (39–39.2)
Race / ethnicity		
NH American Indian or Alaska Native	16 074 (0.45)	38.6 (38–39.3)
NH Asian or Pacific Islander	70 794 (1.97)	24.7 (24.5–24.9)
NH Black or African American	371 523 (10.3)	52.7 (52.5–52.8)
NH White	3 021 305 (84.1)	51.8 (50.6–50.7)
Hispanic or Latino	112 226 (3.12)	20.2 (20.1–20.4)
Age		
<1 year	26 (0.0007)	0 (0–0)
1–4 years	55 (0.001)	0 (0–0)
5–14 years	114 (0.003)	0 (0–0)
15–24 years	572 (0.016)	0.1 (0.1–0.1)
25–34 years	3274 (0.091)	0.4 (0.3–0.4)
35–44 years	37 055 (1.03)	4 (3.9–4)
45–54 years	237 146 (6.59)	25.6 (25.5–25.7)
55–64 years	698 330 (19.4)	91.1 (90.9–91.3)
65–74 years	1 141 438 (31.7)	223.6 (223.2–224)
75–84 years	1 077 556 (29.9)	361 (360.3–361.7)
85+ years	403 959 (11.2)	338 (337–339)
Not stated	52 (0.001)	—
Urbanization		
Metropolitan	2 869 029 (79.7)	46.7 (46.7–46.8)
Non-metropolitan	730 548 (20.3)	55.9 (55.8–56)
Place of death		
Medical facility	1 197 680 (33.3)	—
Decedent’s home	1 465 359 (40.7)	—
Hospice facility	251 370 (6.98)	—
Nursing home/long-term care facility	489 489 (13.6)	—
Others	184 660 (5.13)	—
Unknown	11 019 (0.3)	

AAMR, associated age-adjusted mortality rate; NH, non-Hispanic.

### Annual trends for lung and bronchus cancer-related age-adjusted mortality rates

**Figure 1:**
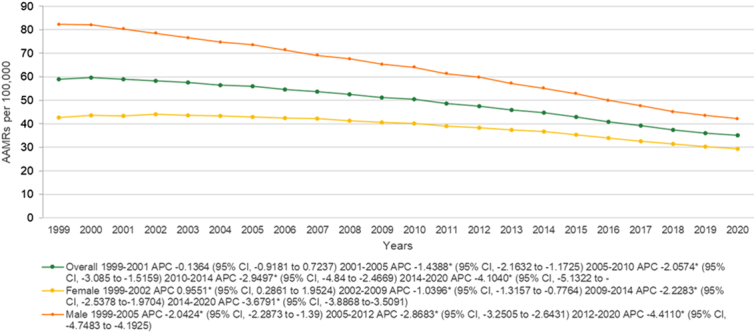
Mortality rates for malignant neoplasm of bronchus and lung, stratified by sex in the population of all ages in the United States between 1999 and 2020, along with the associated age-adjusted mortality rates (AAMRs) per 100 000. The * denotes the annual percentage change (APC) that was found to be statistically significant at α=0.05

The overall AAMR for lung and bronchus cancer was 59.1 (95% CI: 58.8–59.4) in 1999 and decreased to 58.9 (95% CI: 58.6–59.2) in 2001 (APC: −0.1364; 95% CI: −0.9181 to 0.7237). The rates continued to fall after 2001 decreasing to 55.9 (95% CI: 55.6–56.1) by 2005 (APC: −1.4388*; 95% CI: −2.1632 to −1.1725), succeeded by a further downfall at increasing rate becoming 50.5 (95% CI: 50.2–50.7) in 2010 (APC: −2.0574*; 95% CI: −3.085 to −1.5159). In the next decade these trends in AAMR continued, reaching 44.7 (95% CI: 44.4–44.9) by 2014 (APC: −2.9497*; 95% CI: −4.84 to −2.4669) and 35.1 (95% CI: 34.9–35.3) by 2020 (APC: −4.1040*; 95% CI: −5.1322 to −3.125) (Fig. 1, Supplementary Tables 3 and 4, Supplemental Digital Content 1, http://links.lww.com/MS9/A569).

On sensitivity analysis considering neoplasms to be the underlying cause of death mortality rates were relatively constant with a slight decrease between 1999 and 2001 (APC: −0.06; 95% CI: −0.6538 to 0.4642) following which a trend of decreasing mortality at an increasing rate was seen consistently from 2001 to 2005, (APC: −1.37*; 95% CI: −1.8276 to −1.1315) 2005 to 2010 (APC: −2.01*; 95% CI: −2.8237 to −1.7941), 2010 to 2014 (APC: −2.91*; 95% CI:−4.8328 to −2.6414) and 2014 to 2020 (APC: −4.62*; 95% CI: −4.9997 to −4.2676) (Supplementary Figure 1, Supplemental Digital Content 2, http://links.lww.com/MS9/A570)

### Lung and bronchus cancer-related AAMR stratified by sex

Men had consistently higher AAMRs than women during the analyzed years (overall AAMR men: 61.7; 95% CI: 61.6–61.8; women: 38.3; 95% CI: 38.2–38.3). In 1999 men had an AAMR of 82.3 (95% CI: 81.8–82.8) which decreased to 73.6 (95% CI: 73.1–74.1) in 2005 (APC: −2.0424*; 95% CI: −2.2873 to −1.39) followed by a further decrease to 59.8 (95% CI: 59.4–60.2) in 2012 (APC: −2.8683*; 95% CI: −3.2505 to −2.6431) finally going down to 42.2 (95% CI: 41.9–42.5) in 2020 in a steep decrease (APC: −4.4110*; 95% CI: −4.7483 to −4.1925). Contrasting from the men, women AAMR showed an increase from 42.7 (95% CI: 42.3–43) in 1999 to 44 (95% CI: 43.7–44.3) in 2002 (APC: 0.9551*; 95% CI: 0.2861–1.9524) after which a trend for decreasing AAMRs similar to men was continued from 40.7 (95% CI: 40.4–41) in 2009 (APC:−1.0396*; 95% CI: −1.3157 to −0.7764) to 36.7 (95% CI: 36.4–36.9) in 2014 (APC: −2.2283*; 95% CI: −2.5378 to −1.9704) finally decreasing to 29.5 (95% CI: 29.3–29.8) at the end of the study period. (APC: −3.6791*; 95% CI: −3.8868 to −3.5091) (Fig. 1, Supplemental Tables 3 and 4, Supplemental Digital Content 1, http://links.lww.com/MS9/A569).

### Lung and bronchus cancer-related AAMR stratified by race/ethnicity

AAMR stratification according to race/ethnicity revealed that total rates were highest among NH Black or African American patients followed by NH White, NH American Indian or Alaska Native, NH Asian or Pacific Islander with the Hispanic or Latino race having the lowest rates. (Overall AAMR NH Black or African American: 52.7 (95% CI: 52.5–52.8); NH White: 51.8 (95% CI: 50.6–50.7); NH American Indian or Alaska Native: 38.6 (95% CI: 38–39.3); NH Asian or Pacific Islander: 24.7 (95% CI: 24.5–24.9); Hispanic or Latino: 20.2 (95% CI: 20.1–20.4)).

**Figure 2:**
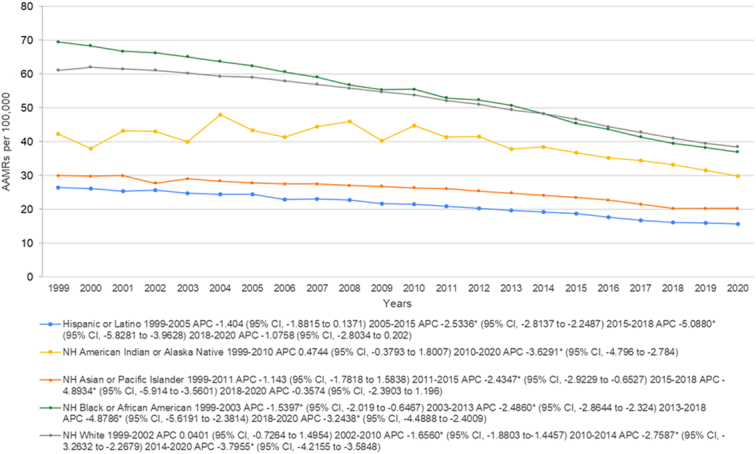
Mortality rates for malignant neoplasm of bronchus and lung, stratified by race in population of all ages in the United States between 1999 and 2020, along with the associated age-adjusted mortality rates (AAMRs) per 100 000. The * denotes the annual percentage change (APC) that was found to be statistically significant at α=0.05.

In summary, the AAMRs of NH Asian or Pacific Islander, Hispanic or Latino, and NH Black or African American patients decreased from the starting point of the study to 2011, 2005, and 2003, respectively. This decreasing trend continued until 2015 for the NH Asian or Pacific Islander and Hispanic or Latino populations and until 2013 for the NH Black or African American, after which all three races saw a steep and similar decrease in rates until 2018 with a continuing decreasing trend being reported for all in between 2018 and 2020. The NH American Indian or Alaskan Native race saw an increase in the trends of mortality between 1999 and 2010 (APC: 0.4744; 95% CI: −0.3793 to 1.8007) followed by a sharp decrease until the end of the study period (APC: −3.6291*; 95% CI: −4.796 to −2.784). Lastly, the NH White population saw an increase from 1999 until 2002 (APC: 0.0401; 95% CI: −0.7264 to 1.4954) followed by a decreasing rate between till 2010 (APC:−1.6560*; 95% CI: −1.8803 to −1.4457), from 2010 to 2014 (APC: −2.7587*; 95% CI: −3.2632 to −2.2679) and from 2014 to 2020 (APC:−3.7955*; 95% CI: −4.2155 to −3.5848) (Fig. 2, Supplemental Tables 3 and 5, Supplemental Digital Content 1, http://links.lww.com/MS9/A569).

### Lung and bronchus cancer-related AAMR stratified by geographic region

**Figure 3:**
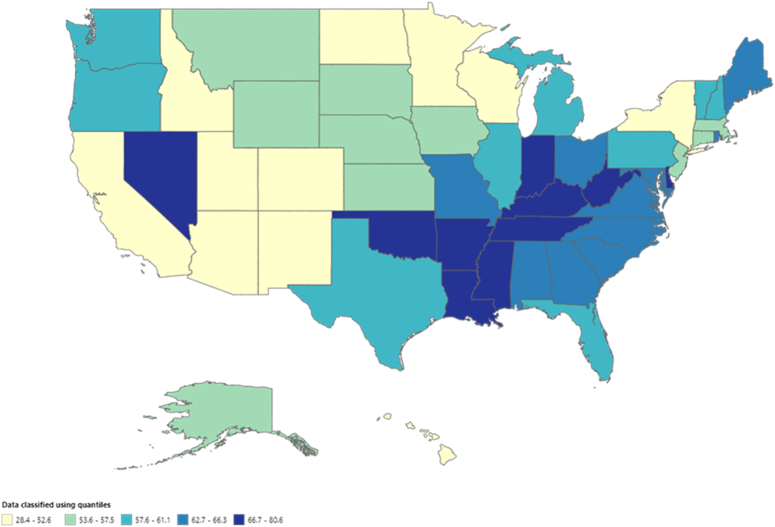
Mortality rates for malignant neoplasm of bronchus and lung, stratified by states in population of all ages in the United States from 1999 to 2020, along with age-adjusted mortality rates per 100 000 among states (ranging from 28.4 to 80.6).

**Figure 4:**
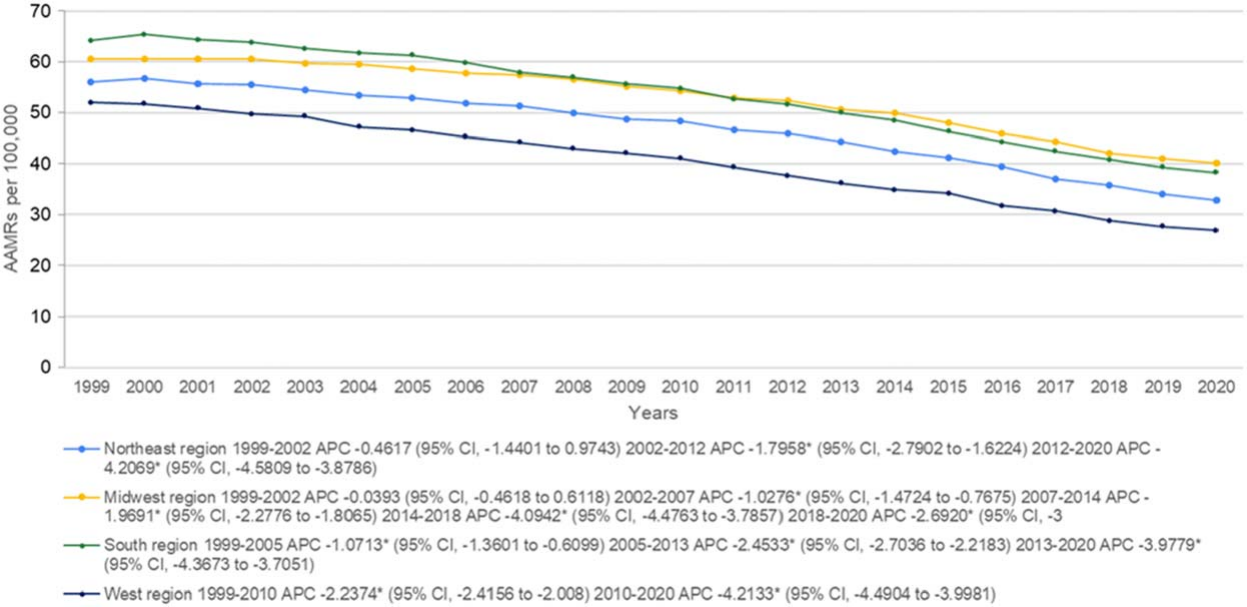
Mortality rates for malignant neoplasm of bronchus and lung, stratified by census region in population of all ages in the United States between 1999 and 2020, along with the associated age-adjusted mortality rates (AAMRs) per 100 000. The * denotes the annual percentage change (APC) that was found to be statistically significant at α=0.05

The states had a wide range of AAMRs starting from 21.5 (95% CI: 21.1–21.9) in Utah to 72.1 (95% CI: 71.6–72.6) in Kentucky with death rates in the top 90^th^ percentile (Arkansas, Kentucky, Mississippi, Tennessee, and West Virginia) being approximately double than those in the lower 10^th^ percentile California, Colorado, Hawaii, New Mexico, Utah (Fig. 3 Supplemental Table 6, Supplemental Digital Content 1, http://links.lww.com/MS9/A569). We also analyzed the mortality rates according to census regions and found the highest AAMRs in the South (AAMR: 52.4; 95% CI: 52.3–52.5), followed by Midwest (AAMR: 52.3; 95% CI: 52.2–52.4), Northeast (AAMR: 46.3; 95% CI: 46.2–46.5), and the West (AAMR: 39.1; 95% CI: 39–39.2). (Fig. 4, Supplemental table 7, Supplemental Digital Content 1, http://links.lww.com/MS9/A569)

**Figure 5:**
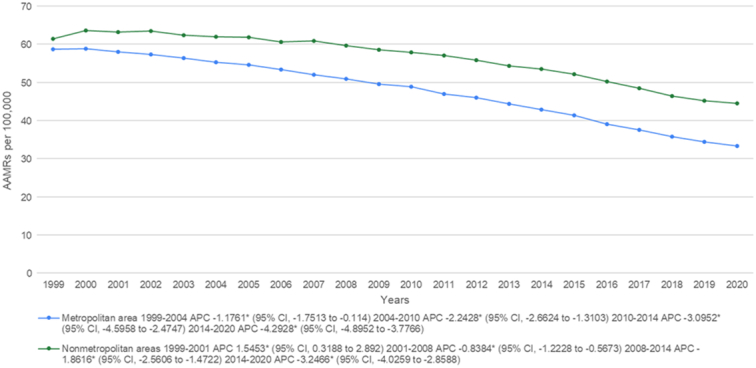
Mortality rates for malignant neoplasm of bronchus and lung, stratified by Urbanization in population of all ages in the United States between 1999 and 2020, along with the associated age-adjusted mortality rates (AAMRs) per 100,000. The * denotes the annual percentage change (APC) that was found to be statistically significant at α=0.05

From 1999 to 2020 non-metropolitan areas had a consistently higher AAMR as compared to metropolitan areas with overall AAMRS being 55.9 (95% CI: 55.8–56) and 46.7 (95% CI: 46.7–46.8), respectively. Initially, non-metropolitan areas saw an increase in mortality from the period between 1999 and 2001 (APC: 1.5453*; 95% CI: 0.3188–2.892) while a decrease was noticed in metropolitan areas from 1999 to 2004 (APC: −1.1761*; 95% CI: −1.7513 to −0.114). After this mortality rates continued to decrease for both groups. For non-metropolitan areas, a slow lowering of mortality was shown from 2001 to 2008 (APC: −0.8384*; 95% CI: −1.2228 to −0.5673) followed by a drastic lowering between 2008 and 2014 (APC: −1.8616*; 95% CI: −2.5606 to −1.4722). Metropolitan areas showed a stark decrease from 2004 to 2010 (APC: −2.2428*; 95% CI: −2.6624 to −1.3103) and from 2010 to 2014 (APC: −3.0952*; 95% CI: −4.5958 to −2.4747). From 2014 to the end of the study period the trend of decreasing mortality rates continued for both strata (non-metropolitan: APC: −3.2466*; 95% CI: −4.0259 to −2.8588, metropolitan: APC: −4.2928*; 95% CI: −4.8952 to −3.7766) (Fig. 5, Supplemental Tables 3 and 8, Supplemental Digital Content 1, http://links.lww.com/MS9/A569).

## Discussion

**Figure 6:**
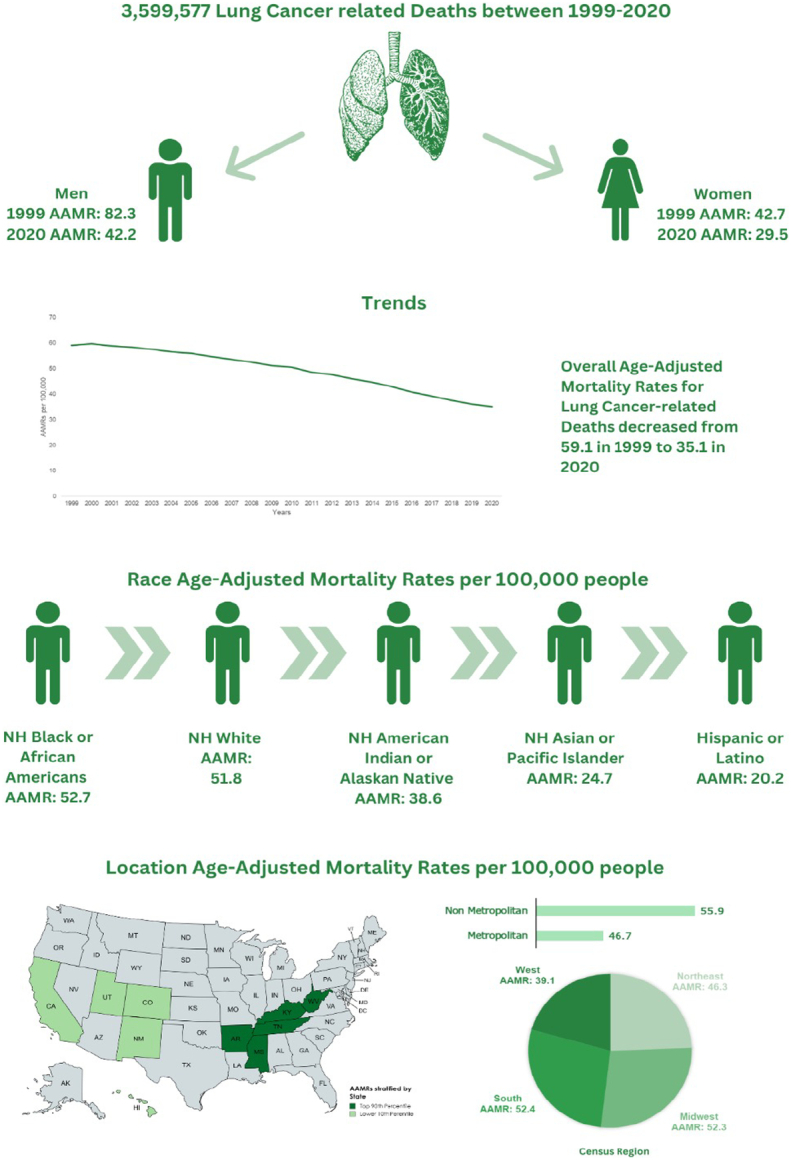
Central Illustration. AAMR, associated age-adjusted mortality rate; NH, non-Hispanic.

In this study, we reported several key findings from a 22-year analysis of lung cancer mortality data from the Centers for Disease Control and Prevention. First, the preliminary findings indicated an overall decline in AAMR for lung cancer from 1999 to 2020. Secondly, men were found to have higher AAMRs compared to women over the study period. While AAMR consistently decreased in men, women reported increased AAMR from 1999 to 2002, which was followed by a similar decline in the succeeding years. Third, the authors also noted that NH Black or African American patients and Hispanic or Latino patients had the highest and lowest total AAMRs, respectively. Compared to Hispanic or Latino, NH Black or African American, and NH Asian or Pacific Islander patients who demonstrated a continued decline in AAMR throughout the study period, NH American Indian or Alaskan Native and NH White patients demonstrated an increase in mortality trends between 1999–2010 and 1999–2002, respectively. Fourth, significant regional differences in lung and bronchus cancer mortality trends were observed, with the South and the West regions having the highest and lowest AAMRs, respectively. It was also noted that the Southern region had the highest AAMR from 1999 to 2010 while the Midwestern region reported the highest AAMR values from 2010 to 2020. (Fig. 6)

Smoking patterns have a profound impact on the mortality trends of lung cancer, stratified by age, sex, socioeconomic status, and race or ethnicity^14^. Individuals belonging to low socioeconomic status have been found to have the highest prevalence of cigarette smoking^15^. The accessibility to lung and bronchus cancer screening is not equitable across states, which can be attributed to state-level differences in Medicaid coverage^16^. Moreover, the under-represented ethnic and racial minorities in the US have a greater likelihood of being uninsured, exacerbating disparities in lung cancer mortality and morbidity^16,17^. African Americans or NH Blacks are known to have the poorest survival rates among other racial or ethnic groups for the majority of cancers including lung cancer^18^. Richmond *et al.* ^19^ demonstrated that Black lung cancer patients had greater unadjusted odds of greater neighborhood deprivation and distant stage diagnosis in the Southeastern region. Racial and ethnic discrimination in the diagnostic experiences have significant ramifications on the treatment of lung cancer^20^.

The sex-based differences in AAMRs of lung and bronchus cancer can be attributed to several mechanisms. Males tend to have an overall greater lung cancer mortality and a higher tumor grade at the time of diagnosis compared to their female counterparts. The sex-relevant considerations are also accounted for outcomes related to chemotherapies and immunotherapies with women and men demonstrating better treatment response, respectively^21^. The incidence of lung cancer is higher among men, however, women are more likely to be diagnosed at a younger age with earlier stage diagnosis^22^. Variability in the smoking patterns elucidates differences in the cancer histological subtype. Adenocarcinoma is more frequent in females compared to males^23^. During the first tobacco epidemic in industrialized countries, lung and bronchus cancer incidence, and mortality increased before declining for males and plateauing for females^24^. In our study, the decline in AAMRs in women was preceded by a rise in lung cancer mortality in women during 1999–2002. This can be explained by the comparatively later initiation of cigarette smoking among women^4,5,25^. The evidence can be forged to tailor public health campaigns, increase access to lung cancer screening programs, adopt sex-specific communication strategies, and address sex disparities in the treatment and prognostic outcomes of lung cancer.

In addition, we also observed higher AAMRs of lung and bronchus cancer in non-metropolitan areas than in metropolitan areas from 1990 to 2020. The mortality rates increased in non-metropolitan areas during 1999–2001, after which a consistent decline was noted. The rate of decline in lung and bronchus cancer mortality was slower during 2001–2008 and relatively rapid during 2008–2014. The rural-urban disparity in lung and bronchus cancer mortality and burden can be represented by the area deprivation index in the rural and urban models^26,27^. Both localized and advanced forms of this cancer have a higher incidence in rural areas, with a comparatively higher disease burden^28^. The differences in lung and bronchus cancer mortality rates across metropolitan and non-metropolitan areas can be accounted for by relatively less access to specialized and routine care as well as worse survival and health outcomes in the latter^29^. The uneven geographic distribution of oncologists among metropolitan and non-metropolitan areas also contributes to the geographic disparities in lung cancer mortality rates^30–33^. The implementation of prevention and control measures including smoking cessation, smoke-free laws, and reduction of exposure to lung carcinogens not only addresses the urban-rural disparities but also decreases lung cancer incidence and mortality rates in non-metropolitan areas^34^.

Regarding the role of lung carcinogens in the environment and the associated risk of lung cancer, a research study demonstrated that non-metropolitan Utah counties had higher rates of incidence of lung cancer compared to metropolitan counties. After adjusting for smoking, the incidence rates were found to be significantly greater in high-radon counties compared to modern radon counties^35^. In addition to this, geographical variations in occupational hazards such as occupational coal and arsenic exposure. Local mitigation strategies and policies, however, determine the radon and arsenic levels in the environment and individual exposure levels^7^. Several other factors that contribute to the elevated lung cancer mortality rate include comorbidities and the disease stage. According to the literature, the majority of lung cancer patients visit hospitals when the disease is in advanced stages. Moreover, patients residing in rural areas have a higher mortality rate due to a lack of adequate healthcare facilities^36^. Lastly, the degree of adherence of individuals to screening programs and the type of screening programs also influence the incidence of lung cancer and the mortality benefits. Centralized surveillance programs tend to have a higher adherence rate compared to decentralized surveillance programs^37^.

## Limitations

While the study has made significant contributions to understanding lung and bronchus cancer mortality trends and associated variables, it has several limitations. Firstly, the reliance on ICD-10 codes and death certificates is related to considerable variation across different healthcare settings, giving rise to misclassification bias and subsequent misinterpretation of data leading to the omission of lung and bronchus cancer as a cause of death. We also acknowledge that our dataset does not include information related to cancer biomarkers, smoking, lung and bronchus cancer prevalence in the family, therapeutic interventions, and duration between diagnosis and death of lung cancer patients. Data pertaining to the treatment of lung and bronchus neoplasms using medical therapy is unavailable. Additionally, data concerning the socioeconomic factors influencing health, which could impact access to care, is also lacking.

## Conclusion

We thoroughly examined and presented the Centers for Disease Control and Prevention mortality data from 1999 to 2020. Our findings indicate a decreasing trend in the AAMR for lung and bronchus cancer in the overall U.S. population. The highest AAMRs were observed among non-Hispanic Black or African American adults, males, residents of the southern regions in earlier years while midwestern later on, and those living in non-metropolitan areas across all age groups in the U.S. population. The study emphasizes the importance of addressing lung and bronchus cancer screening disparities and undertaking public health measures to regulate the risk factors and precipitating events.

## Ethical approval

This study was exempted from the institutional review board’s approval because it uses publicly available data that is de-identified.

## Consent

Informed consent was not required for this review.

## Source of funding

None.

## Author contribution

H.A.U.R.: conceptualization, data curation, formal analysis, writing—original draft. M.A.A.F.: data curation, writing—original draft. A.S.: conceptualization, writing—original draft. S.K.: data curation, writing—original draft. A.R.: data curation, writing—original draft. S.R.: data curation, writing—original draft. D.A.: data curation, writing—original draft. R.D.: writing—original draft. A.K.: writing—original draft

## Conflicts of interest disclosure

The authors declare that they have no known competing financial interests or personal relationships that could have appeared to influence the work reported in this paper.

## Research registration unique identifying number (UIN)

This isn’t needed as this paper is a comprehensive review and not a systematic review or meta-analysis.

## Guarantor

Hafsah Alim Ur Rahman and Muhammad Ahmed Ali Fahim.

## Data availability statement

The dataset supporting the conclusions of this article is included in this article.

## Provenance and peer review

The paper was not invited.

## Supplementary Material

## References

[1] Siegel RL Giaquinto AN Jemal A. Cancer statistics, 2024. CA Cancer J Clin 2024;74:12–49. 38230766 10.3322/caac.21820

[2] American Cancer Society. (2024) Key Statistics for Lung Cancer. https://www.cancer.org/cancer/types/lung-cancer/about/key-statistics.html

[3] Islami F Goding Sauer A Miller KD. Proportion and number of cancer cases and deaths attributable to potentially modifiable risk factors in the United States. CA Cancer J Clin 2018;68:31–54. 29160902 10.3322/caac.21440

[4] Harris JE. Cigarette smoking among successive birth cohorts of men and women in the United States during 1900–1980. J Natl Cancer Inst 1983;71:473–479. 6577223

[5] Jemal A Ma J Rosenberg PS. Increasing lung cancer death rates among young women in southern and midwestern states. J Clin Oncol 2012;30:2739. 22734032 10.1200/JCO.2012.42.6098 PMC3402885

[6] Huang J Deng Y Tin MS. Distribution, risk factors, and temporal trends for lung cancer incidence and mortality: a global analysis. Chest 2022;161:1101–1111. 35026300 10.1016/j.chest.2021.12.655

[7] Shreves AH Buller ID Chase E. Geographic patterns in US lung cancer mortality and cigarette smoking. Cancer Epidemiol Biomarkers Prev 2023;32:193–201. 36413442 10.1158/1055-9965.EPI-22-0253 PMC9905286

[8] John U Hanke M. Lung cancer mortality and years of potential life lost among males and females over six decades in a country with high smoking prevalence: an observational study. BMC Cancer 2015;15:876. 26553055 10.1186/s12885-015-1807-7 PMC4640109

[9] Centers for Disease Control and Prevention About Multiple Cause of Death, 1999-2020. Accessed 1 Mar 2024. https://wonder.cdc.gov/mcd-icd10.html.

[10] Aggarwal R Chiu N Loccoh EC. Rural-Urban disparities: diabetes, hypertension, heart disease, and stroke mortality among Black and White Adults, 1999-2018. J Am Coll Cardiol 2021;77:1480–1481. 33736831 10.1016/j.jacc.2021.01.032 PMC8210746

[11] Ingram DD Franco SJ. 2013 NCHS Urban-Rural Classification Scheme for Counties. Vital Health Stat 2014;2:1–73. 24776070

[12] Anderson RN Rosenberg HM. Age standardization of death rates: implementation of the year 2000 standard. Natl vital Stat reports from Centers Dis Control Prev Natl Cent Heal Stat Natl Vital Stat Syst 1998;47:1–16; 20. 9796247

[13] National Cancer Institute Joinpoint Help System - Surveillance Research Program.

[14] Jeon J Inoue-Choi M Mok Y. Mortality relative risks by smoking, race/ethnicity, and education. Am J Prev Med 2023;64:S53–S62. 36775754 10.1016/j.amepre.2022.12.006 PMC11186465

[15] Jamal A Phillips E Gentzke AS. Current cigarette smoking among adults - United States, 2016. MMWR Morb Mortal Wkly Rep 2018;67:53–59. 29346338 10.15585/mmwr.mm6702a1 PMC5772802

[16] Rivera MP Katki HA Tanner NT. Addressing disparities in lung cancer screening eligibility and healthcare access. an Official American Thoracic Society Statement. Am J Respir Crit Care Med 2020;202:e95–e112. 33000953 10.1164/rccm.202008-3053ST PMC7528802

[17] Haddad DN Sandler KL Henderson LM. Disparities in lung cancer screening: a review. Ann Am Thorac Soc 2020;17:399–405. 32017612 10.1513/AnnalsATS.201907-556CME PMC7175982

[18] Prosper A Brown K Schussel B. Lung cancer screening in African Americans: the time to act is now. Radiol Imaging Cancer 2020;2:e200107. 33778737 10.1148/rycan.2020200107 PMC7983712

[19] Richmond J Murray MH Milder CM. Racial disparities in lung cancer stage of diagnosis among adults living in the Southeastern United States. Chest 2023;163:1314–1327. 36435265 10.1016/j.chest.2022.11.025 PMC10206508

[20] Thuo N Martins T Manley E. Factors leading to disparity in lung cancer diagnosis among black/African American communities in the USA: a qualitative study. BMJ Open 2023;13:e073886. 10.1136/bmjopen-2023-073886 PMC10619042 37899158

[21] May L Shows K Nana-Sinkam P. Sex differences in lung cancer. Cancers (Basel) 2023;15:3111. 37370722 10.3390/cancers15123111 PMC10296433

[22] Stabellini N Bruno DS Dmukauskas M. Sex differences in lung cancer treatment and outcomes at a large hybrid academic-community practice. JTO Clin Res Rep 2022;3:100307. 35400080 10.1016/j.jtocrr.2022.100307 PMC8983352

[23] Kiyohara C Ohno Y. Sex differences in lung cancer susceptibility: a review. Gend Med 2010;7:381–401. 21056866 10.1016/j.genm.2010.10.002

[24] Mederos N Friedlaender A Peters S. Gender-specific aspects of epidemiology, molecular genetics and outcome: lung cancer. ESMO Open 2020;5:e000796. 33148544 10.1136/esmoopen-2020-000796 PMC7643520

[25] Siegel RL Miller KD Wagle NS. Cancer statistics, 2023. Ca Cancer J Clin 2023;73:17–48. 36633525 10.3322/caac.21763

[26] Fairfield KM Black AW Ziller EC. Area deprivation index and rurality in relation to lung cancer prevalence and mortality in a rural state. JNCI Cancer Spectr 2020;4:pkaa011. 32676551 10.1093/jncics/pkaa011 PMC7353952

[27] Henley SJ Anderson RN Thomas CC. Invasive cancer incidence, 2004-2013, and deaths, 2006-2015, in nonmetropolitan and metropolitan counties—United States. Morb Mortal Wkly Rep Surveill Summ (Washington, DC 2002) 2017;66:1–13. 10.15585/mmwr.ss6614a1 PMC5879727 28683054

[28] Zahnd WE Fogleman AJ Jenkins WD. Rural-urban disparities in stage of diagnosis among cancers with preventive opportunities. Am J Prev Med 2018;54:688–698. 29550163 10.1016/j.amepre.2018.01.021

[29] Cornelius SL Shaefer AP Wong SL. Comparison of US oncologist rurality by practice setting and patients served. JAMA Netw Open 2024;7:e2350504. 38180759 10.1001/jamanetworkopen.2023.50504 PMC10770776

[30] Aboagye JK Kaiser HE Hayanga AJ. Rural-urban differences in access to specialist providers of colorectal cancer care in the United States: a physician workforce issue. JAMA Surg 2014;149:537–543. 24740165 10.1001/jamasurg.2013.5062

[31] Levit LA Byatt L Lyss AP. Closing the rural cancer care gap: three institutional approaches. JCO Oncol Pract 2020;16:422–430. 32574128 10.1200/OP.20.00174

[32] Unger JM Moseley A Symington B. Geographic distribution and survival outcomes for rural patients with cancer treated in clinical trials. JAMA Netw Open 2018;1:e181235. 30646114 10.1001/jamanetworkopen.2018.1235 PMC6324281

[33] Hung P Deng S Zahnd WE. Geographic disparities in residential proximity to colorectal and cervical cancer care providers. Cancer 2020;126:1068–1076. 31702829 10.1002/cncr.32594

[34] O’Neil ME Henley SJ Rohan EA. Lung cancer incidence in nonmetropolitan and metropolitan counties—United States, 2007-2016. MMWR Morb Mortal Wkly Rep 2019;68:993–998. 31697655 10.15585/mmwr.mm6844a1 PMC6837473

[35] Ou JY Fowler B Ding Q. A statewide investigation of geographic lung cancer incidence patterns and radon exposure in a low-smoking population. BMC Cancer 2018;18:1–8. 29385999 10.1186/s12885-018-4002-9 PMC5793382

[36] Tesfaw LM Dessie ZG Mekonnen Fenta H. Lung cancer mortality and associated predictors: systematic review using 32 scientific research findings. Front Oncol 2023;13:1308897. 38156114 10.3389/fonc.2023.1308897 PMC10754488

[37] Sakoda LC Rivera MP Zhang J. Patterns and factors associated with adherence to lung cancer screening in diverse practice settings. JAMA Netw Open 2021;4:e218559. 33929519 10.1001/jamanetworkopen.2021.8559 PMC8087957

