# Supplementary material for: Investigating sex, race, and geographic disparities in bronchus and lung cancer mortality in the United States: a comprehensive longitudinal study (1999–2020) utilizing CDC WONDER data
Source: Ann Med Surg (Lond). 2024 Aug 7;86(9):5361–9. doi: 10.1097/MS9.0000000000002387 (PMC11374286; doi:10.1097/MS9.0000000000002387)
Supplement: Supplementary file 1 [file ms9-86-5361-s001.docx]

**Supplementary Information**

**Supplementary Table 1**: Lung Cancer related Deaths, Stratified by Sex and Race, in the United States, 1999 to 2020

| **Deaths** | | | | | | | | | |
| --- | --- | --- | --- | --- | --- | --- | --- | --- | --- |
| **Year** | **Overall** | **Women** | **Men** | **NH White** | **NH Black or African American** | **NH Asian or Pacific Islander** | **NH American Indian or Alaska Native** | **Hispanic or Latino** | **Population** |
| 1999 | 162190 | 66592 | 95598 | 138751 | 16729 | 2035 | 512 | 3735 | 279040168 |
| 2000 | 165436 | 68891 | 96545 | 141828 | 16712 | 2147 | 490 | 3837 | 281421906 |
| 2001 | 165940 | 69581 | 96359 | 142006 | 16677 | 2289 | 569 | 4006 | 284968955 |
| 2002 | 167553 | 71517 | 96036 | 143070 | 16977 | 2270 | 586 | 4246 | 287625193 |
| 2003 | 167690 | 72055 | 95635 | 142901 | 17060 | 2495 | 566 | 4308 | 290107933 |
| 2004 | 167590 | 72388 | 95202 | 142451 | 17094 | 2609 | 707 | 4419 | 292805298 |
| 2005 | 168840 | 73110 | 95730 | 143336 | 17205 | 2692 | 676 | 4698 | 295516599 |
| 2006 | 168174 | 73344 | 94830 | 142682 | 17175 | 2813 | 658 | 4590 | 298379912 |
| 2007 | 167967 | 74245 | 93722 | 142185 | 17119 | 2936 | 717 | 4837 | 301231207 |
| 2008 | 168021 | 74072 | 93949 | 142035 | 16863 | 3043 | 758 | 5043 | 304093966 |
| 2009 | 167294 | 74256 | 93038 | 141127 | 16988 | 3140 | 715 | 5041 | 306771529 |
| 2010 | 167612 | 74521 | 93091 | 140654 | 17374 | 3237 | 809 | 5200 | 308745538 |
| 2011 | 165891 | 74025 | 91866 | 138843 | 17213 | 3442 | 772 | 5375 | 311591917 |
| 2012 | 166975 | 74775 | 92200 | 139154 | 17496 | 3549 | 830 | 5513 | 313914040 |
| 2013 | 165592 | 74546 | 91046 | 137597 | 17593 | 3658 | 774 | 5633 | 316128839 |
| 2014 | 164883 | 74663 | 90220 | 136689 | 17335 | 3830 | 831 | 5786 | 318857056 |
| 2015 | 163199 | 74107 | 89092 | 134839 | 16926 | 4005 | 836 | 6021 | 321418820 |
| 2016 | 158766 | 72444 | 86322 | 130805 | 16750 | 4033 | 847 | 5885 | 323127513 |
| 2017 | 156254 | 71718 | 84536 | 128707 | 16354 | 4074 | 865 | 5839 | 325719178 |
| 2018 | 152977 | 70591 | 82386 | 125863 | 16035 | 3975 | 868 | 5903 | 327167434 |
| 2019 | 150790 | 69559 | 81231 | 123415 | 15994 | 4156 | 851 | 6072 | 328239523 |
| 2020 | 149943 | 69283 | 80660 | 122367 | 15854 | 4366 | 837 | 6239 | 329484123 |
| **Total** | **3,599,577** | **1,590,283** | **2,009,294** | **3,021,305** | **371,523** | **70,794** | **16,074** | **112,226** | **6746356647** |

*NH, non-Hispanic.*

**Supplementary Table 2;** Lung Cancer related Mortality, Stratified by Place of Death in the United States, 1999 to 2020

| **Deaths** | | | | | | |
| --- | --- | --- | --- | --- | --- | --- |
| **Year** | **Medical Facility** | **Nursing Home** | **Hospices** | **Home** | **Other** | **Unknown** |
|  |  |  |  |  |  |  |
| 1999 | 68056 | 23999 | Missing | 62286 | 7818 | 31 |
| 2000 | 67276 | 25047 | Missing | 64497 | 8564 | 52 |
| 2001 | 67032 | 24949 | Missing | 64452 | 9472 | 35 |
| 2002 | 66904 | 25324 | Missing | 64945 | 10350 | 30 |
| 2003 | 64677 | 24951 | 860 | 65725 | 10973 | 504 |
| 2004 | 62874 | 25062 | 1465 | 66177 | 11566 | 446 |
| 2005 | 61902 | 25085 | 4765 | 66741 | 9913 | 434 |
| 2006 | 59711 | 24958 | 6469 | 67084 | 9441 | 511 |
| 2007 | 58967 | 24961 | 8452 | 66076 | 9295 | 216 |
| 2008 | 56410 | 24412 | 10548 | 64809 | 8599 | 3243 |
| 2009 | 52974 | 24120 | 11335 | 65090 | 8933 | 4842 |
| 2010 | 53375 | 23403 | 13829 | 67394 | 9507 | 104 |
| 2011 | 50884 | 22734 | 15246 | 67560 | 9385 | 82 |
| 2012 | 49925 | 21067 | 17741 | 68796 | 9349 | 97 |
| 2013 | 48029 | 20971 | 17606 | 69169 | 9724 | 93 |
| 2014 | 46765 | 20477 | 20687 | 69770 | 7055 | 129 |
| 2015 | 45645 | 20067 | 21951 | 69625 | 5870 | 41 |
| 2016 | 45078 | 19489 | 21683 | 66843 | 5637 | 36 |
| 2017 | 44287 | 18855 | 21272 | 66282 | 5531 | 27 |
| 2018 | 43542 | 18103 | 20915 | 64829 | 5565 | 23 |
| 2019 | 43286 | 17942 | 20323 | 63525 | 5694 | 20 |
| 2020 | 40081 | 13513 | 16223 | 73684 | 6419 | 23 |
| **Total** | 1197680 | 489489 | 251370 | 1465359 | 184660 | 11019 |

**Supplementary Table 3** Annual percent change (APC) of Lung Cancer related Age-Adjusted Mortality Rates per 100,000 in the United States, 1999 to 2020

| **Year Interval** | **APC (95% CI)** |
| --- | --- |
| **Overall** | |
| 1999-2001 | -0.1364 (-0.9181 to 0.7237) |
| 2001-2005 | -1.4388* (-2.1632 to -1.1725) |
| 2005-2010 | -2.0574* (-3.085 to -1.5159) |
| 2010-2014 | -2.9497* (-4.84 to -2.4669) |
| 2014-2020 | -4.1040* (-5.1322 to -3.125) |
| **Male** | |
| 1999-2005 | -2.0424* (-2.2873 to -1.39) |
| 2005-2012 | -2.8683* (-3.2505 to -2.6431) |
| 2012-2020 | -4.4110* (-4.7483 to -4.1925) |
| **Female** | |
| 1999-2002 | 0.9551* (0.2861 to 1.9524) |
| 2002-2009 | -1.0396* (-1.3157 to -0.7764) |
| 2009-2014 | -2.2283* (-2.5378 to-1.9704) |
| 2014-2020 | -3.6791* (-3.8868 to-3.5091) |
| **NH White** | |
| 1999-2002 | 0.0401 (-0.7264 to 1.4954) |
| 2002-2010 | -1.6560* (-1.8803 to-1.4457) |
| 2010-2014 | -2.7587* (-3.2632 to -2.2679) |
| 2014-2020 | -3.7955* (-4.2155 to -3.5848) |
| **NH Black or African American** | |
| 1999-2003 | -1.5397* (-2.019 to -0.6467) |
| 2003-2013 | -2.4860* (-2.8644 to -2.324) |
| 2013-2018 | -4.8786* (-5.6191 to -2.3814) |
| 2018-2020 | -3.2438* (-4.4888 to -2.4009) |
| **NH American Indian or Alaska Native** | |
| 1999-2010 | 0.4744 (-0.3793 to 1.8007) |
| 2010-2020 | -3.6291* (-4.796 to -2.784) |
| **NH Asian or Pacific Islander** | |
| 1999-2011 | -1.143 (-1.7818 to 1.5838) |
| 2011-2015 | -2.4347* (-2.9229 to -0.6527) |
| 2015-2018 | -4.8934* (-5.914 to -3.5601) |
| 2018-2020 | -0.3574 (-2.3903 to 1.196) |
| **Hispanic or Latino** | |
| 1999-2005 | -1.404 (-1.8815 to 0.1371) |
| 2005-2015 | -2.5336* (-2.8137 to -2.2487) |
| 2015-2018 | -5.0880* (-5.8281 to -3.9628) |
| 2018-2020 | -1.0758 (-2.8034 to 0.202) |
| **Nonmetropolitan areas** | |
| 1999-2001 | 1.5453* (0.3188 to 2.892) |
| 2001-2008 | -0.8384* (-1.2228 to -0.5673) |
| 2008-2014 | -1.8616* (-2.5606 to -1.4722) |
| 2014-2020 | -3.2466* (-4.0259 to -2.8588) |
| **Metropolitan area** | |
| 1999-2004 | -1.1761* (-1.7513 to -0.114) |
| 2004-2010 | -2.2428* (-2.6624 to -1.3103) |
| 2010-2014 | -3.0952* (-4.5958 to -2.4747) |
| 2014-2020 | -4.2928* (-4.8952 to -3.7766) |
| **Northeast region** | |
| 1999-2002 | -0.4617 (-1.4401 to 0.9743) |
| 2002-2012 | -1.7958* (-2.7902 to -1.6224) |
| 2012-2020 | -4.2069* (-4.5809 to -3.8786) |
| **Midwest region** | |
| 1999-2002 | -0.0393 (-0.4618 to 0.6118) |
| 2002-2007 | -1.0276* (-1.4724 to -0.7675) |
| 2007-2014 | -1.9691* (-2.2776 to -1.8065) |
| 2014-2018 | -4.0942* (-4.4763 to -3.7857) |
| 2018-2020 | -2.6920* (-3.3979 to -2.2203) |
| **South region** | |
| 1999-2005 | -1.0713* (-1.3601 to -0.6099) |
| 2005-2013 | -2.4533* (-2.7036 to -2.2183) |
| 2013-2020 | -3.9779* (-4.3673 to -3.7051) |
| **West region** | |
| 1999-2010 | -2.2374* (-2.4156 to -2.008) |
| 2010-2020 | -4.2133* (-4.4904 to -3.9981) |

*APC = annual percent change; NH = non-Hispanic; * Indicates that the annual percentage change* *(APC) is significantly different from zero at α = 0.05. AAMR = age-adjusted mortality rate.*

**Supplementary Table 4:** Overall and Sex‐Stratified Lung Cancer related Age-Adjusted Mortality Rates per 100,000 in the United States, 1999 to 2020

| **Age-Adjusted Rate (95% CI)** | | | |
| --- | --- | --- | --- |
| **Year** | **Male** | **Female** | **Overall** |
| 1999 | 82.3 (81.8-82.8) | 42.7 (42.3-43) | 59.1 (58.8-59.4) |
| 2000 | 82.1 (81.6-82.6) | 43.7 (43.4-44) | 59.7 (59.4-60) |
| 2001 | 80.4 (79.9-80.9) | 43.5 (43.2-43.8) | 58.9 (58.6-59.2) |
| 2002 | 78.5 (78-79) | 44 (43.7-44.3) | 58.4 (58.1-58.7) |
| 2003 | 76.6 (76.1-77.1) | 43.6 (43.3-44) | 57.5 (57.2-57.7) |
| 2004 | 74.7 (74.3-75.2) | 43.3 (42.9-43.6) | 56.5 (56.2-56.8) |
| 2005 | 73.6 (73.1-74.1) | 42.9 (42.6-43.3) | 55.9 (55.6-56.1) |
| 2006 | 71.4 (70.9-71.9) | 42.4 (42.1-42.7) | 54.7 (54.4-54.9) |
| 2007 | 69.1 (68.6-69.5) | 42.2 (41.9-42.5) | 53.6 (53.3-53.9) |
| 2008 | 67.6 (67.1-68) | 41.3 (41-41.6) | 52.5 (52.2-52.7) |
| 2009 | 65.3 (64.9-65.7) | 40.7 (40.4-41) | 51.2 (50.9-51.4) |
| 2010 | 64.1 (63.7-64.6) | 40.2 (39.9-40.5) | 50.5 (50.2-50.7) |
| 2011 | 61.3 (60.9-61.7) | 39.1 (38.8-39.3) | 48.6 (48.4-48.9) |
| 2012 | 59.8 (59.4-60.2) | 38.4 (38.2-38.7) | 47.6 (47.4-47.9) |
| 2013 | 57.2 (56.9-57.6) | 37.5 (37.2-37.7) | 46 (45.8-46.3) |
| 2014 | 55.1 (54.7-55.4) | 36.7 (36.4-36.9) | 44.7 (44.4-44.9) |
| 2015 | 52.8 (52.5-53.2) | 35.4 (35.2-35.7) | 43 (42.8-43.2) |
| 2016 | 50 (49.7-50.3) | 33.9 (33.6-34.1) | 40.9 (40.7-41.1) |
| 2017 | 47.7 (47.4-48.1) | 32.7 (32.4-32.9) | 39.2 (39.1-39.4) |
| 2018 | 45.2 (44.9-45.5) | 31.4 (31.2-31.7) | 37.5 (37.3-37.7) |
| 2019 | 43.6 (43.3-43.9) | 30.3 (30-30.5) | 36.1 (35.9-36.3) |
| 2020 | 42.2 (41.9-42.5) | 29.5 (29.3-29.8) | 35.1 (34.9-35.3) |
| **Total** | **61.7 (61.6-61.8)** | **38.3 (38.2-38.3)** | **48.3 (48.3-48.4)** |

| **Age-Adjusted Rate (95% CI)** | | | | | |
| --- | --- | --- | --- | --- | --- |
| **Year** | **NH White** | **NH Black or African American** | **NH American Indian or Alaska Native** | **Hispanic or Latino** | **NH Asian or Pacific Islander** |
| 1999 | 61.1 (60.7-61.4) | 69.5 (68.4-70.6) | 42.2 (38.4-46.1) | 26.4 (25.5-27.3) | 29.9 (28.5-31.2) |
| 2000 | 62 (61.7-62.3) | 68.3 (67.3-69.4) | 37.9 (34.4-41.4) | 26.1 (25.3-27) | 29.8 (28.5-31.2) |
| 2001 | 61.5 (61.1-61.8) | 66.7 (65.7-67.7) | 43.2 (39.5-47) | 25.3 (24.4-26.1) | 29.9 (28.7-31.2) |
| 2002 | 61.1 (60.7-61.4) | 66.2 (65.2-67.2) | 43.1 (39.4-46.7) | 25.6 (24.8-26.4) | 27.7 (26.5-28.9) |
| 2003 | 60.2 (59.9-60.5) | 65.1 (64.1-66.1) | 39.9 (36.4-43.4) | 24.8 (24-25.6) | 29 (27.8-30.1) |
| 2004 | 59.3 (59-59.7) | 63.7 (62.7-64.7) | 47.9 (44.2-51.6) | 24.4 (23.6-25.1) | 28.3 (27.2-29.5) |
| 2005 | 59 (58.7-59.3) | 62.4 (61.4-63.3) | 43.3 (39.9-46.8) | 24.5 (23.8-25.3) | 27.8 (26.7-28.9) |
| 2006 | 57.9 (57.6-58.2) | 60.6 (59.6-61.5) | 41.3 (37.9-44.6) | 22.9 (22.2-23.6) | 27.5 (26.4-28.5) |
| 2007 | 56.9 (56.6-57.2) | 59.1 (58.2-60) | 44.5 (41-47.9) | 23 (22.3-23.7) | 27.5 (26.5-28.5) |
| 2008 | 55.8 (55.5-56.1) | 56.8 (56-57.7) | 45.9 (42.5-49.4) | 22.8 (22.2-23.5) | 27 (26-28) |
| 2009 | 54.7 (54.4-55) | 55.3 (54.4-56.2) | 40.2 (37-43.3) | 21.6 (21-22.2) | 26.8 (25.8-27.8) |
| 2010 | 53.8 (53.5-54.1) | 55.5 (54.6-56.3) | 44.7 (41.4-48) | 21.5 (20.9-22.1) | 26.3 (25.3-27.2) |
| 2011 | 52.1 (51.8-52.4) | 52.9 (52.1-53.8) | 41.4 (38.3-44.5) | 20.9 (20.3-21.5) | 26.1 (25.2-27) |
| 2012 | 51 (50.8-51.3) | 52.3 (51.5-53.1) | 41.5 (38.5-44.5) | 20.3 (19.8-20.9) | 25.4 (24.5-26.3) |
| 2013 | 49.4 (49.2-49.7) | 50.7 (49.9-51.4) | 37.8 (35-40.6) | 19.7 (19.2-20.3) | 24.8 (23.9-25.6) |
| 2014 | 48.2 (48-48.5) | 48.2 (47.5-49) | 38.4 (35.6-41.1) | 19.2 (18.7-19.7) | 24.1 (23.3-24.8) |
| 2015 | 46.6 (46.4-46.9) | 45.4 (44.7-46.1) | 36.7 (34.1-39.3) | 18.8 (18.3-19.3) | 23.5 (22.7-24.2) |
| 2016 | 44.4 (44.2-44.6) | 43.7 (43-44.3) | 35.2 (32.7-37.7) | 17.7 (17.2-18.1) | 22.7 (22-23.4) |
| 2017 | 42.8 (42.6-43.1) | 41.4 (40.7-42) | 34.4 (32-36.8) | 16.7 (16.3-17.1) | 21.5 (20.9-22.2) |
| 2018 | 41 (40.8-41.2) | 39.5 (38.9-40.1) | 33.2 (30.9-35.5) | 16.1 (15.7-16.5) | 20.2 (19.6-20.8) |
| 2019 | 39.5 (39.3-39.7) | 38.2 (37.6-38.8) | 31.5 (29.3-33.6) | 16 (15.6-16.4) | 20.2 (19.6-20.8) |
| 2020 | 38.5 (38.3-38.7) | 36.9 (36.3-37.5) | 29.8 (27.7-31.9) | 15.6 (15.2-16) | 20.2 (19.6-20.8) |
| **Total** | 51.8 (50.6-50.7) | 52.7 (52.5-52.8) | 38.6 (38-39.3) | 20.2 (20.1-20.4) | 24.7 (24.5-24.9) |

**Supplementary Table 5**: Lung Cancer–related Age-Adjusted Mortality Rates per 100,000, Stratified by Race in the United States, 1999 to 2020

*NH: Non-Hispanic.*

**Supplementary Table 6:** Lung Cancer–related Age-Adjusted Mortality Rates per 100,000, Stratified by States in the United States, 1999 to 2020

| **State** | **Age-Adjusted Rate (95% CI)** |
| --- | --- |
| Alabama | 58 (57.5-58.4) |
| Alaska | 48 (46.6-49.4) |
| Arizona | 39.1 (38.8-39.4) |
| Arkansas | 63.8 (63.3-64.4) |
| California | 37.5 (37.4-37.7) |
| Colorado | 34.6 (34.2-35) |
| Connecticut | 43.7 (43.2-44.1) |
| Delaware | 54.1 (53.1-55) |
| District of Columbia | 44.6 (43.4-45.7) |
| Florida | 47.2 (47-47.4) |
| Georgia | 50.7 (50.4-51) |
| Hawaii | 34.8 (34.1-35.4) |
| Idaho | 39.6 (38.9-40.2) |
| Illinois | 50.5 (50.3-50.8) |
| Indiana | 59.3 (58.9-59.7) |
| Iowa | 49.8 (49.3-50.3) |
| Kansas | 50.1 (49.6-50.7) |
| Kentucky | 72.1 (71.6-72.6) |
| Louisiana | 57.8 (57.3-58.2) |
| Maine | 56.5 (55.7-57.2) |
| Maryland | 48.5 (48.1-48.9) |
| Massachusetts | 47.7 (47.3-48) |
| Michigan | 53 (52.7-53.3) |
| Minnesota | 42.9 (42.5-43.3) |
| Mississippi | 63.4 (62.8-64) |
| Missouri | 58.1 (57.8-58.5) |
| Montana | 44.8 (44-45.6) |
| Nebraska | 46.4 (45.7-47) |
| Nevada | 49.3 (48.7-49.8) |
| New Hampshire | 49.8 (49.1-50.6) |
| New Jersey | 43.8 (43.5-44.1) |
| New Mexico | 33.3 (32.8-33.8) |
| New York | 43.4 (43.2-43.6) |
| North Carolina | 53.8 (53.5-54.1) |
| North Dakota | 43.3 (42.3-44.3) |
| Ohio | 56.8 (56.6-57.1) |
| Oklahoma | 60.6 (60.1-61.1) |
| Oregon | 48 (47.5-48.4) |
| Pennsylvania | 49.9 (49.6-50.1) |
| Rhode Island | 52.5 (51.7-53.4) |
| South Carolina | 53.7 (53.3-54.2) |
| South Dakota | 46.4 (45.5-47.4) |
| Tennessee | 62.4 (62-62.8) |
| Texas | 44.9 (44.7-45.1) |
| Utah | 21.5 (21.1-21.9) |
| Vermont | 50.2 (49.1-51.3) |
| Virginia | 49.1 (48.8-49.4) |
| Washington | 46.6 (46.3-46.9) |
| West Virginia | 65.3 (64.6-66) |
| Wisconsin | 46.3 (45.9-46.6) |
| Wyoming | 40.9 (39.8-42) |

**Supplementary Table 7:** Lung Cancer–related Age-Adjusted Mortality Rates per 100,000, Stratified by Census Region in the United States, 1999 to 2020

| **Census Region** | **Year** | **Age-Adjusted Rate (95% CI)** |
| --- | --- | --- |
| Northeast | 1999 | 56 (55.4-56.6) |
| Northeast | 2000 | 56.8 (56.1-57.4) |
| Northeast | 2001 | 55.7 (55.1-56.3) |
| Northeast | 2002 | 55.5 (54.9-56.1) |
| Northeast | 2003 | 54.4 (53.8-55) |
| Northeast | 2004 | 53.4 (52.8-53.9) |
| Northeast | 2005 | 52.8 (52.2-53.4) |
| Northeast | 2006 | 51.9 (51.3-52.5) |
| Northeast | 2007 | 51.4 (50.8-51.9) |
| Northeast | 2008 | 49.9 (49.4-50.5) |
| Northeast | 2009 | 48.7 (48.1-49.2) |
| Northeast | 2010 | 48.4 (47.8-48.9) |
| Northeast | 2011 | 46.6 (46.1-47.1) |
| Northeast | 2012 | 46 (45.5-46.5) |
| Northeast | 2013 | 44.3 (43.8-44.8) |
| Northeast | 2014 | 42.5 (42-43) |
| Northeast | 2015 | 41.3 (40.8-41.8) |
| Northeast | 2016 | 39.5 (39-40) |
| Northeast | 2017 | 37 (36.5-37.4) |
| Northeast | 2018 | 35.9 (35.5-36.3) |
| Northeast | 2019 | 34.1 (33.7-34.5) |
| Northeast | 2020 | 32.9 (32.5-33.3) |
| Northeast | **Total** | 46.3 (46.2-46.5) |
| Midwest | 1999 | 60.5 (59.9-61.1) |
| Midwest | 2000 | 60.6 (60-61.2) |
| Midwest | 2001 | 60.5 (60-61.1) |
| Midwest | 2002 | 60.5 (59.9-61) |
| Midwest | 2003 | 59.7 (59.1-60.2) |
| Midwest | 2004 | 59.5 (59-60.1) |
| Midwest | 2005 | 58.6 (58-59.2) |
| Midwest | 2006 | 57.7 (57.1-58.2) |
| Midwest | 2007 | 57.5 (56.9-58) |
| Midwest | 2008 | 56.5 (55.9-57) |
| Midwest | 2009 | 55.1 (54.5-55.6) |
| Midwest | 2010 | 54.3 (53.8-54.8) |
| Midwest | 2011 | 52.8 (52.3-53.4) |
| Midwest | 2012 | 52.3 (51.8-52.8) |
| Midwest | 2013 | 50.6 (50.1-51.1) |
| Midwest | 2014 | 49.9 (49.5-50.4) |
| Midwest | 2015 | 48 (47.5-48.4) |
| Midwest | 2016 | 45.9 (45.4-46.3) |
| Midwest | 2017 | 44.4 (43.9-44.8) |
| Midwest | 2018 | 42.1 (41.6-42.5) |
| Midwest | 2019 | 41 (40.6-41.5) |
| Midwest | 2020 | 40.1 (39.6-40.5) |
| Midwest | **Total** | 52.3 (52.2-52.4) |
| South | 1999 | 64.2 (63.7-64.7) |
| South | 2000 | 65.4 (64.9-65.9) |
| South | 2001 | 64.3 (63.8-64.8) |
| South | 2002 | 63.8 (63.3-64.3) |
| South | 2003 | 62.6 (62.1-63.1) |
| South | 2004 | 61.7 (61.2-62.2) |
| South | 2005 | 61.3 (60.9-61.8) |
| South | 2006 | 59.8 (59.3-60.3) |
| South | 2007 | 57.9 (57.5-58.3) |
| South | 2008 | 56.9 (56.5-57.3) |
| South | 2009 | 55.6 (55.1-56) |
| South | 2010 | 54.8 (54.4-55.2) |
| South | 2011 | 52.7 (52.2-53.1) |
| South | 2012 | 51.6 (51.2-52) |
| South | 2013 | 50 (49.6-50.4) |
| South | 2014 | 48.5 (48.1-48.8) |
| South | 2015 | 46.3 (45.9-46.6) |
| South | 2016 | 44.3 (43.9-44.6) |
| South | 2017 | 42.5 (42.2-42.9) |
| South | 2018 | 40.8 (40.4-41.1) |
| South | 2019 | 39.3 (39-39.6) |
| South | 2020 | 38.3 (38-38.6) |
| South | **Total** | 52.4 (52.3-52.5) |
| West | 1999 | 52 (51.4-52.6) |
| West | 2000 | 51.7 (51.1-52.3) |
| West | 2001 | 50.8 (50.2-51.4) |
| West | 2002 | 49.7 (49.2-50.3) |
| West | 2003 | 49.3 (48.7-49.8) |
| West | 2004 | 47.2 (46.7-47.7) |
| West | 2005 | 46.6 (46-47.1) |
| West | 2006 | 45.3 (44.8-45.9) |
| West | 2007 | 44.2 (43.7-44.7) |
| West | 2008 | 43 (42.5-43.5) |
| West | 2009 | 42.1 (41.6-42.6) |
| West | 2010 | 41.1 (40.6-41.5) |
| West | 2011 | 39.3 (38.9-39.8) |
| West | 2012 | 37.7 (37.3-38.1) |
| West | 2013 | 36.2 (35.8-36.7) |
| West | 2014 | 34.9 (34.5-35.3) |
| West | 2015 | 34.2 (33.8-34.6) |
| West | 2016 | 31.8 (31.4-32.2) |
| West | 2017 | 30.7 (30.3-31) |
| West | 2018 | 28.8 (28.4-29.1) |
| West | 2019 | 27.7 (27.4-28.1) |
| West | 2020 | 26.9 (26.5-27.2) |
| West | **Total** | 39.1 (39-39.2) |
| **Total** | **Total** | 48.3 (48.3-48.4) |

**Supplementary Table 8:** Lung Cancer–related Age-Adjusted Mortality Rates per 100,000, Stratified by Urban-Rural Classification in the United States, 1999 to 2020

| **Age-Adjusted Rate (95% CI)** | | |
| --- | --- | --- |
| **Year** | **Metropolitan** | **Nonmetropolitan** |
| 1999 | 58.7 (58.3-59) | 61.4 (60.7-62.1) |
| 2000 | 58.8 (58.5-59.2) | 63.6 (62.9-64.3) |
| 2001 | 58 (57.6-58.3) | 63.2 (62.5-63.9) |
| 2002 | 57.3 (57-57.6) | 63.4 (62.8-64.1) |
| 2003 | 56.4 (56.1-56.7) | 62.3 (61.7-63) |
| 2004 | 55.3 (55-55.6) | 61.9 (61.3-62.6) |
| 2005 | 54.6 (54.3-54.9) | 61.8 (61.1-62.4) |
| 2006 | 53.4 (53.1-53.7) | 60.6 (59.9-61.2) |
| 2007 | 52 (51.7-52.3) | 60.9 (60.2-61.5) |
| 2008 | 50.9 (50.7-51.2) | 59.7 (59-60.3) |
| 2009 | 49.6 (49.4-49.9) | 58.6 (57.9-59.2) |
| 2010 | 48.9 (48.7-49.2) | 57.9 (57.3-58.5) |
| 2011 | 46.9 (46.6-47.2) | 57 (56.4-57.6) |
| 2012 | 46 (45.7-46.2) | 55.8 (55.2-56.4) |
| 2013 | 44.3 (44.1-44.6) | 54.3 (53.7-54.8) |
| 2014 | 42.9 (42.7-43.1) | 53.5 (52.9-54) |
| 2015 | 41.3 (41-41.5) | 52.1 (51.5-52.6) |
| 2016 | 39.1 (38.9-39.3) | 50.2 (49.6-50.7) |
| 2017 | 37.5 (37.3-37.7) | 48.4 (47.9-49) |
| 2018 | 35.8 (35.6-36) | 46.4 (45.9-46.9) |
| 2019 | 34.4 (34.2-34.6) | 45.2 (44.7-45.8) |
| 2020 | 33.3 (33.1-33.5) | 44.5 (44-45.1) |
| **Total** | **46.7 (46.7-46.8)** | **55.9 (55.8-56)** |
