# Supplementary material for: Investigating sex, race, and geographic disparities in bronchus and lung cancer mortality in the United States: a comprehensive longitudinal study (1999–2020) utilizing CDC WONDER data
Source: Ann Med Surg (Lond). 2024 Aug 7;86(9):5361–9. doi: 10.1097/MS9.0000000000002387 (PMC11374286; doi:10.1097/MS9.0000000000002387)
Supplement: Supplementary file 2 [file ms9-86-5361-s002.docx]

**Supplementary Information**

**Supplementary Figure 1:** Sensitivity Analysis for Malignant neoplasm of bronchus and lung, in the United States between 1999 and 2020, according to neoplasms, along with the associated age-adjusted mortality rates (AAMRs) per 100,000.

**
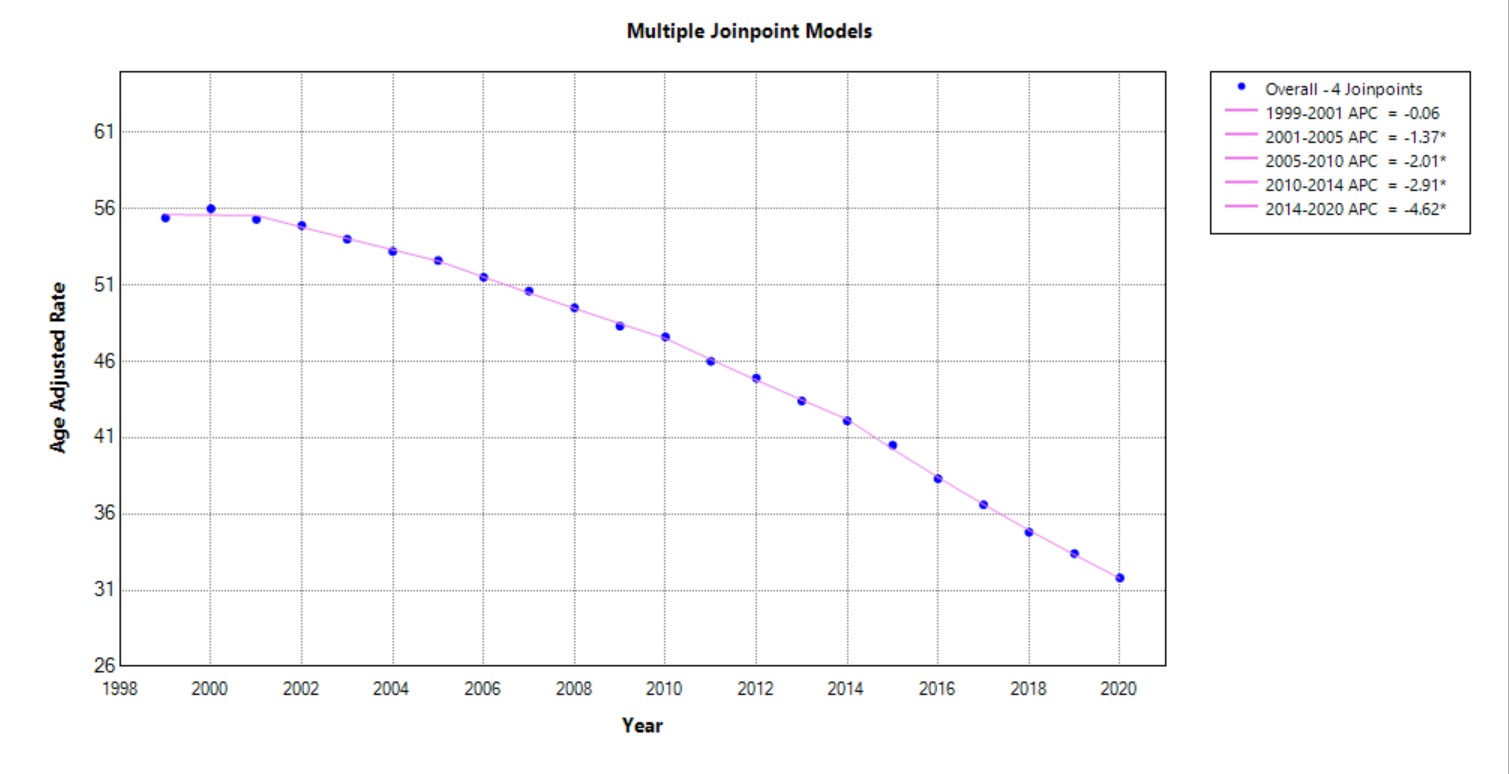
**
